# Effect of Different Ambient Temperatures on Reproductive Outcome and Stress Level of Lactating Females in Two Mouse Strains

**DOI:** 10.3390/ani12162141

**Published:** 2022-08-20

**Authors:** Thomas Kolbe, Caroline Lassnig, Andrea Poelzl, Rupert Palme, Kerstin E. Auer, Thomas Rülicke

**Affiliations:** 1 Biomodels Austria, University of Veterinary Medicine Vienna, 1210 Vienna, Austria; 2 Department IFA-Tulln, University of Natural Resources and Life Sciences, 1180 Vienna, Austria; 3 Institute of Animal Breeding and Genetics, University of Veterinary Medicine Vienna, 1210 Vienna, Austria; 4 Unit of Physiology, Pathophysiology and Experimental Endocrinology, University of Veterinary Medicine Vienna, 1210 Vienna, Austria; 5 Institute of in vivo and in vitro Models, University of Veterinary Medicine Vienna, 1210 Vienna, Austria; 6 Department of Biomedical Sciences, University of Veterinary Medicine Vienna, 1210 Vienna, Austria

**Keywords:** ambient temperature, homeothermy, breeding mice, stress, glucocorticoids, lactation

## Abstract

**Simple Summary:**

The optimal temperature for laboratory mice has been under discussion for some time. Current standard temperature is 20 °C–24 °C but it has been suggested to elevate the standard to 30 °C, which is the thermoneutral zone for mice. In this study, the effect of different cage temperatures (20 °C, 25 °C, 30 °C) on reproduction and stress hormone metabolite excretion was evaluated in lactating females of two commonly used mouse strains. Pup loss was higher, and weights of mothers and pups were reduced at 30 °C compared to the lower temperatures. In addition, pups showed increased tail length at weaning under the high temperature (30 °C). There was no difference in stress hormone metabolite excretion in mice between temperature groups. We could not show any detrimental effects of the lower or higher cage temperature on stress hormone metabolite excretion, but found decreased reproductive outcome under the higher temperature.

**Abstract:**

Ambient temperature is an important non-biotic environmental factor influencing immunological and oncological parameters in laboratory mice. It is under discussion which temperature is more appropriate and whether the commonly used room temperature in rodent facilities of about 21 °C represents a chronic cold stress or the 30 °C of the thermoneutral zone constitutes heat stress for the animals. In this study, we selected the physiological challenging period of lactation to investigate the influence of a cage temperature of 20 °C, 25 °C, and 30 °C, respectively, on reproductive performance and stress hormone levels in two frequently used mouse strains. We found that B6D2F1 hybrid mothers weaned more pups compared to C57BL/6N mothers, and that the number of weaned pups was reduced when mothers of both strains were kept at 30 °C. Furthermore, at 30 °C, mothers and pups showed reduced body weight at weaning and offspring had longer tails. Despite pronounced temperature effects on reproductive parameters, we did not find any temperature effects on adrenocortical activity in breeding and control mice. Independent of the ambient temperature, however, we found that females raising pups showed elevated levels of faecal corticosterone metabolites (FCMs) compared to controls. Peak levels of stress hormone metabolites were measured around birth and during the third week of lactation. Our results provide no evidence of an advantage for keeping lactating mice in ambient temperatures near the thermoneutral zone. In contrast, we found that a 30 °C cage temperature during lactation reduced body mass in females and their offspring and declined female reproductive performance.

## 1. Introduction

Aiming to study thermoregulatory behavior in mice Gordon and Coworkers [1] started a discussion about the optimal ambient temperature, which culminated in a widely noticed publication of Hylander and Repasky [2]. The authors emphasized in their paper the different results of immunological and oncological studies when conducted at 20 °C or at 30 °C. Consequently, the results of studies on mouse models for human diseases performed at 20–26 °C standard ambient temperature were questioned and considered to be temperature biased, because of low reproducibility if performed under higher ambient temperatures [3,4,5,6]. It is generally accepted that room temperature can influence experimental results like many other biotic and non-biotic environmental factors [7]. However, some of the reported effects related to ambient temperature emerge only when mice were heated up to a body temperature of 39–40 °C for 6 h [8,9,10,11,12] or to 42 °C for 40 min [13].

Although a comprehensive analysis about the appropriate ambient temperature for laboratory mice in experiments is still missing, the call for housing laboratory mice in their thermoneutral zone as standard ambient temperature arose. The thermoneutral zone is defined as a temperature range in which the general metabolism of the organism, in the absence of any physical activity, generates sufficient heat as a byproduct of the continually ongoing metabolism to maintain the predetermined body temperature [14]. Thermal physiology of nocturnal mice seems to be different between dark and light periods. Influenced by the circadian rhythm two discrete ambient temperatures are proposed as thermoneutral points (TNP): ~29 °C in the light phase and ~33 °C in the dark phase [15]. In initial tests mice preferred to stay in warmer areas of experimental settings even if nesting material was provided. These thermoregulatory experiments were conducted using a copper pipe with a wire mesh inside [1] or an aluminum channel [16], heated at one side, cooled at the opposite side. This setup led to the assumption that mice prefer an ambient temperature near their homeothermic temperature of 30 °C. In later studies, a more common laboratory mouse environment was used [17,18]. By offering bedding and nesting material, it became obvious that the preferred ambient temperature depends on the activity of mice and the amount and quality of nesting material [19,20,21,22,23]. With enough and useful nesting material mice can prevent their body from cooling down during resting periods [24]. Depending on activity, the body core temperature can change between 36 °C and 37 °C [25]. Also, the homeothermic zone seems to be more a temperature point than a zone and varies about 4 °C across the day. Temperatures below this homeothermic point lead to increased energy expenditures, whereas temperatures above lead to a rise in body temperature [15].

For a naked human being the thermoneutral zone is similar to that of mice and ranges between 28 °C and 29 °C [26]. But as soon as the human body is covered with light clothing (e.g., long sleeved shirt or blouse and light trousers) this range drops down to 23–25 °C [27] or to 15–25 °C with regular clothing (e.g., a business suit) [26]. Offering mice bedding and nesting material for insulation could have a comparable effect as clothing in humans. Thus, mice can adapt to different ambient temperatures, given that sufficient bedding and nesting material is available. Moreover, they are able to adjust their body core temperature depending on activity and environmental conditions and are even able to survive ambient temperatures from −10 °C to 32 °C [28]. Interestingly, this characteristic seems to be dependent on sex, strain, age or an interaction of these variables. For example, when kept at ambient temperatures either at 20 °C or 30 °C, 6 months old C57BL/6 females showed a subcutaneous temperature difference of 0.5 °C [25]. In contrast, 2 months old CD1 males kept at these two temperatures showed a 2 °C difference [13], and no difference in body temperature was found in 6 weeks old BALB/c females either at 20 °C or 30 °C ambient temperature [11]. Even between phases of activity and inactivity mouse body temperature differed in about 1 °C [24,29,30,31,32]. Another study found that at 20 °C, mouse body temperature was not influenced by the presence or absence of nesting material, only food consumption was increased in the absence of nesting material [20]. Age [33] and strain [34] can influence experimental data that are collected at homeothermic (30 °C) or common facility temperatures (20 °C).

However, which temperature mice prefer in regard of their wellbeing, is a still open question. Tumor bearing mice, i.e., morbid animals, preferred higher temperatures, likely because their thermoregulation is potentially already defective [35]. In preference tests healthy mice spent more time in warmer surroundings when they were inactive, i.e., slept or rested, or when only cage bedding was available [16]. When nesting material was offered and mice had the possibility to carry it into cages with different ambient temperatures, they allocated it in cooler cages and used it for nest building to insulate themselves while resting [17]. However, others report that even when nesting material was provided, adult female mice showed a preference for warmer environments, especially in their inactive phase, compared to male mice of the same age [36]. Possible effects of ambient temperatures on animal welfare have been addressed [29,30,32] and reproductive parameters like birth rate, weaning rate and embryo quality were investigated in relation to this environmental factor in mice [21,22,37,38]. In addition, increased sleeping apneas [39] and behavioral changes, such as increased male aggression [40] were reported for mice in studies with higher ambient temperature.

Toth and co-workers [32] were the first to investigate the impact of ambient temperatures on animal welfare by measuring faecal corticosterone metabolites (FCMs) in mice kept at different room temperatures. Measuring FCMs is a proven non-invasive method to evaluate the animals’ stress hormone levels [41,42,43,44]. In the above mentioned study no difference in FCM concentration was found in adult C57BL/6J female mice when maintained at ambient temperatures of 22 °C, 26 °C or 30 °C, though the applied method to measure FCM was not validated [32,44].

There are no studies to our knowledge, regarding the optimal ambient temperature for the wellbeing of lactating mice. Lactation is a highly demanding metabolic process [45,46] accompanied by considerable metabolic heat production as a by-product. The time of lactation determines pup quality and survival, and understanding the effect of ambient temperatures on lactating mothers and their pups will not only improve animal keeping conditions, but help to optimize animal breeding.

In this study we investigated the impact of different ambient temperatures (20 °C, 25 °C, and 30 °C) on the reproductive performance and stress level of female mice from two different strains. We used C57BL/6N mice as this strain is the most commonly used inbred strain, and B6D2F1 hybrids as this strain shows high fertility during lactation. We measured the impact of ambient temperatures on food consumption, amount of voided faeces and individual body weight of lactating females. The reproductive performance was assessed by comparing the number of implantation sites, the number of born and weaned offspring, as well as pup weight and offspring tail length at weaning. Additionally, we determined FCM levels in mice to assess whether 20 °C are experienced as ‘cold stress’, as postulated in some publications, or 30 °C as ‘heat stress’.

## 2. Materials and Methods

### 2.1. Animals and Husbandry Conditions

A total of 30 male C57BL/6N (referred to as B6N) and 30 male B6D2F1 at the age of 8 weeks and 60 female B6N and 60 female B6D2F1 at the age of 6 weeks were purchased from Janvier Laboratories, Laval, France. Mice were specific pathogen free (SPF) according to FELASA recommendations and maintained in a barrier rodent facility. Groups of 3 to 4 females and single males were housed 2 weeks in type II Macrolon^®^ cages for acclimatization. The cages were lined with 120 g bedding (Lignocel^®^ Select, 3.5–4.5 mm poplar chips, Rettenmaier KG, Austria) and enriched with nesting material (8 g Arbocel^®^ Crinklets natural, Rettenmaier KG, Vienna, Austria; 1 g (two pieces) PurZellin, Paul Hartmann GesmbH, Austria) (photoperiod 12L:12D). Food (V1534 for males, non-pregnant females and females without pups, V1124 for pregnant females and females with pups, Ssniff Spezialdiaeten GmbH, Germany) and tap water in 250 mL bottles were available ad libitum.

Experimental procedures were discussed and approved by the Ethics and Welfare Committee of the University of Veterinary Medicine, Vienna and the national authority (Austrian Federal Ministry of Education, Science and Research) according to §§ 26ff. of the Animal Experiments Act, Tierversuchsgesetz 2012–TVG 2012 under license number BMBWF-68–205/0162-V/3b/2019.

### 2.2. Experimental Temperature Groups

**Figure 1:**
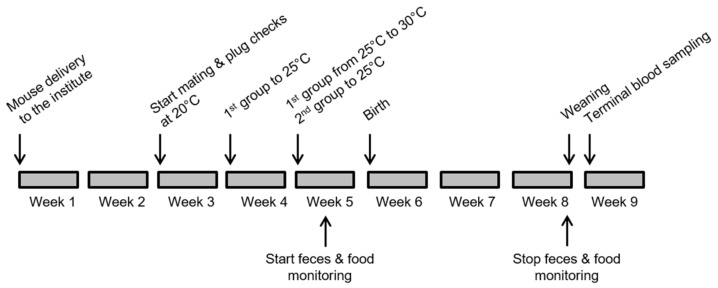
Experimental time schedule. Schematic description of the experimental manipulations and sample collections performed throughout the experiment.

The aim of the study was to investigate the effect of different ambient temperatures on lactating female mice and their offspring. During the period of adaptation, all animals were housed at the standard 20 °C cage temperature in our facility. It is important to mention here that we used open top cages, a type of housing that is increasingly rare in breeding facilities, and that temperatures in IVC cages are usually 1–2 °C higher compared to open top cages. To induce pregnancy, females were mated bigamously with males of the same strain and checked daily for the presence of a vaginal plug, which confirmed mating. Every day plug positive females were separated and re-housed in strain-specific groups of 3 to 4. Within 4 days, 37 females per strain were plug positive. These females were randomly assigned (13/12/12) to one of the three temperature groups (20 °C/25 °C/30 °C). In addition, 8 B6N and 8 B6D2F1 plug negative or non-mated females, and 8 B6N and 8 B6D2F1 males of the same age were used as controls for each temperature group. In previous studies it has been shown that laboratory mice are able to cope with the selected ambient temperatures. Seven days after the detection of a vaginal plug the group assigned to the 30 °C cage temperature was transferred to another room with 25 °C for seven additional days before being relocated to another room with 30 °C for the last week of pregnancy, birth, and lactation. This stepwise adaptation to the highest selected ambient temperature was applied to reduce possible stress induced by a drastic increase in ambient temperature. The second group, which was assigned to 25 °C cage temperature, was transferred to a 25 °C room 14 days after vaginal plug detection. The third group stayed in the room with 20 °C cage temperature from the beginning and remained there until the end of the experiment (Figure 1). Consequently, at least one week before the expected birth date all experimental and control animals were in rooms with their assigned cage temperature. No other experimental treatments were applied to the animals. All temperature rooms were identical in shape and equipment and differed only in their room temperatures. We expected pup births about 20 days after plug detection. Four days before expected parturition all animals (pregnant and control mice) were separated into single cages. Because birth took place between 18 to 21 days after plug detection, the exact number of days under increased ambient temperatures before parturition differed slightly.

### 2.3. Experimental Measurements

Cage temperature was measured with five temperature loggers per room (DS1921G, Thermochron, OnSolution Pty Ltd., Baulkham Hills, NSW, Australia) deposited in the bedding of 5 cages with mice on different rack levels. Measurements were recorded every two hours. Humidity was recorded twice a day (at weekends only once) with standard hygrometers at 3 different positions in the room. We monitored pregnancies, and recorded the day of birth, the number of pups per litter at birth and at weaning. Over a period of 4 weeks, i.e., from last week of pregnancy until weaning, we measured animal food consumption once a week for 24 h. Therefore, we took the weight of the food in the hopper at the beginning and at the end of the 24 h period without spillage correction. Tail length of pups was measured on day 21 *post-partum* with a digital calliper. We calculated the relative tail length of pups (=tail length/body mass) to correct for strain specific size differences. Body weight of adults was measured at weaning using an electronic scale (Model 440–47N, Kern, Germany). The scale was adjusted with a reference weight. For male and female controls the day of the first weaning in the experimental groups was used as reference. Individual pup weight of all litters was taken on the same day at a pup age between 16 to 21 days to assess intra-litter variation. For assessment of inter-litter variation the whole litter weight was taken at weaning (d 20) and mean body mass was calculated by dividing the whole litter weight by the number of pups.

### 2.4. Implantation Sites

In order to evaluate the number of born pups in relation to the number of implanted embryos we dissected the uteri of breeding females *post mortem* at the end of the study. This is important as a daily check of pups might miss pups which were eaten within the first 24 h after birth. We opened the uterine horns with scissors and stained the implantation sites with a few drops of 10% ammonia solution [47]. After a few minutes of reaction implantation sites, visible as dark spots, were counted.

### 2.5. Analysis of Faecal Corticosterone Metabolites and Plasma Corticosterone

We sampled faeces daily at the same time to determine faecal corticosterone metabolites (FCMs) during the overnight activity phase in all mice. We started sample collection a few days before females gave birth and continued daily until the weaning of pups. At the day of birth samples were taken after birth. At weaning samples were taken directly after weaning. Excretion of stress metabolites occur 4–8 h after a stress stimulus, thus stress of birth can only be measured in sample of the following day and any stress of weaning remained undetected since weaning was the last sampling day. Due to the high sample number only two samples per mouse and week were analysed. The first two time points were 1–3 days prior to birth (because of differing birth dates). The third sample time point was for mothers on the day of birth and for corresponding controls at the same day. Sample time points 4–9 followed in 3–4 days intervals. Sample collection for controls occurred at the same dates. The last time point was the day of weaning (Figure 1).

For sample collection, mice were put individually into clean pipette boxes for 15 min and fresh faeces were collected. If the amount of voided faeces during this time period was insufficient for analysis, respective mice were put into clean type III cages without bedding and the collection interval was prolonged for up to 30 min. Samples were stored at −20 °C and FCMs were determined according to a routinely used protocol. Briefly, dried and homogenised faeces were weighted and mixed with 80% methanol, centrifuged, the supernatant was diluted and an aliquot was analysed in a well-established and validated 5α-pregnane-3β,11β,21-triol-20-one enzyme immunoassay (EIA) [48,49].

Additionally, once a week a 24 h sample collection was performed. Therefore, animals were transferred to a fresh cage and after 24 h bedding and faeces were collected and frozen. As voided faeces were mixed with the fresh bedding we sorted the faecal pellets later by hand before weighing. The total amounts of excreted faeces within 24 h was re-corded in mice between temperature groups to be able to account for differences in food consumption and of droppings, respectively. If mice consume less food and secrete fewer droppings, this might lead to increased concentrations of FCMs per gram faeces and vice versa.

After weaning, all mice including respective controls were sacrificed by cervical dislocation and blood was collected by heart puncture. Serum was prepared and analysed for blood corticosterone. Plasma samples were extracted with diethyl-ether and analysed with a previously described corticosterone EIA [50].

### 2.6. Statistical Procedures

Statistical analyses were performed with IBM SPSS Statistics 24. To assess how cage temperature affected female reproduction we performed different models. First, we run a Generalized Linear Model (GLM) with a binomial distribution where we included the incidence of pregnancies as the dependent variable and we run a GLM with a Poisson distribution, where we included the number of implantation sites, litter size at birth and at weaning as dependent variables. Finally, we performed Linear Models (LM), where we included litter weight at weaning, female body mass at weaning, mean pup body mass and pup tail lengths as dependent variables. Mouse strain and cage temperature were always included as fixed factors to all models and Least Significant Difference (LSD) Test was applied as post-hoc test to assess differences between temperature groups. We further tested whether the variation in individual pup body mass (SDs) within litters differed depending on their cage temperature with a Kruskal Wallis Test.

To assess how the experimental manipulations affected FCM levels, food consumption and faeces production over the course of the experiment, we performed repeated measures ANOVAs. We included individual FCM levels, the calculated amount of daily food consumption, and the repeatedly recorded daily faeces production as dependent variables, cage temperature, strain, animal sex and female breeding status as fixed factors. To assess differences within groups Least Significant Difference (LSD) Test was applied as post-hoc test. Finally, we also assessed plasma corticosterone levels with a LM where we included cage temperature and mouse strain as fixed factors.

We tested in all models if model assumptions were fulfilled and transformed data if necessary.

## 3. Results

### 3.1. Cage Temperature and Room Humidity

Experimentally intended cage temperatures were constantly maintained. Relative humidity decreased with increasing ambient temperatures. At 30 °C air temperature humidity was comparatively more fluctuating, but at all times within the range of 30% to 50% (see Supplementary Materials Table S1). 

### 3.2. Reproductive Parameters

Out of 74 females with a mating plug and additional two females without a detectable plug, 54 (71.1%) became pregnant and 22 plugged females (28.9%) did not show any signs of gestation. Pregnancy rates were not affected by cage temperature (χ^2^ = 4.24, *p* = 0.120), but were significantly higher in B6D2F1 compared to B6N females (χ^2^ = 11.90, *p* = 0.001; Table 1).

Females gave birth to an average of 7.5 pups per litter and litter size at birth did not differ between cage temperature (GLM: χ^2^ = 0.29, *p* = 0.863) or strain (GLM: χ^2^ = 1.63, *p* = 0.202). Similarly, the number of female implantation sites (mean: 8.2) did not differ between cage temperature (GLM: χ^2^ = 0.09, *p* = 0.957) or strain (GLM: χ^2^ = 0.16, *p* = 0.694). Rare cases of dead newborns were exclusively detected within 24 h after parturition and were not further considered in the study.

**Figure 2:**
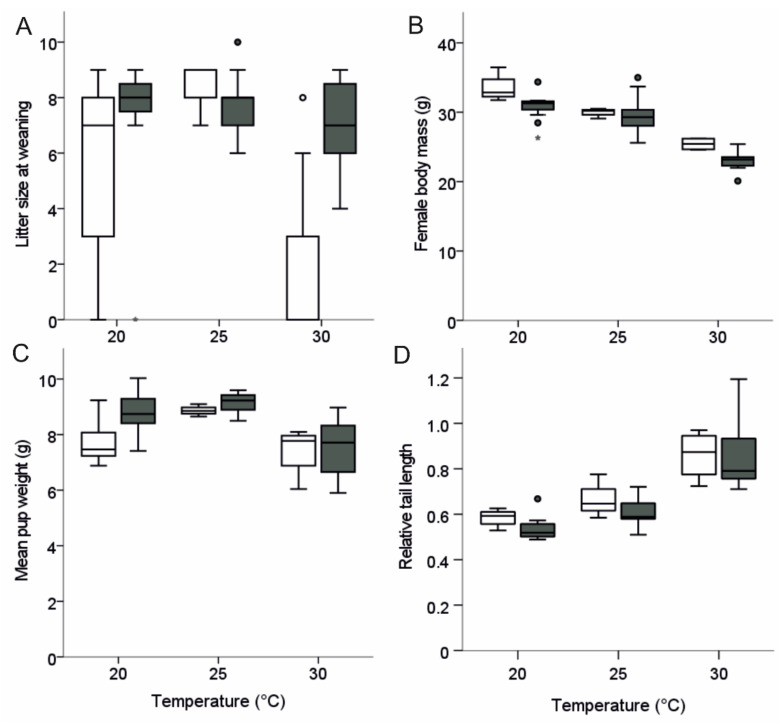
Boxplot of (**A**) litter size at weaning, (**B**) body mass of reproducing females at weaning, (**C**) mean pup weight at weaning, and (**D**) relative tail length of weaned pups of B6N (white boxes) and B6D2F1 (grey boxes) females kept at 20 °C, 25 °C, and 30 °C. Dot = mild outlier (Q1 − 1.5 × IQ, or Q3 + 1.5 × IQ), asterisk = extreme outlier (Q1 − 3 × IQ, or Q3 + 3 × IQ).

We found that cage temperature had a significant effect on the number of pups weaned (GLM: χ^2^ = 7.19, *p* = 0.027; Figure 2A), and females kept at 30 °C weaned fewer pups compared to females kept at either 20 °C (*p* = 0.042) or 25 °C (*p* = 0.002). No difference was found in the number of pups weaned in females kept at 20 °C compared to 25 °C (*p* = 0.197). Also, B6D2F1 females weaned significantly more pups compared to B6N females (GLM: χ^2^ = 14.8, *p* < 0.001; Figure 2A). The number of litters corresponds to the number of females giving birth (Table 1).

### 3.3. Weight and Tail Length

Similar to litter size at weaning, we also observed that litter weight at weaning was significantly affected by cage temperature (F = 17.71, *p* < 0.001). Females kept at 30 °C showed significantly lower litter weaning weights compared to females kept at 25 °C (*p* < 0.001) or 20 °C (*p* < 0.001). No difference in litter weaning weight was detected between females kept at 25 °C or 20 °C (*p* = 0.218). In addition, B6D2F1 females weaned significantly heavier litters compared to B6N females (F = 7.94, *p* = 0.007), though the body weight of B6D2F1 mothers were significantly lower at weaning than that of B6N mothers (F = 8.88, *p* = 0.005; Figure 2B). Even more important, female body mass of both strains was also affected by cage temperature (F = 70.64, *p* < 0.001; Figure 2B) and significantly declined with increasing temperatures (all post-hoc tests *p* ≤ 0.011; see Supplement Materials Figure S1).

Mean pup body mass also differed significantly between cage temperatures (F = 13.39, *p* < 0.001; Figure 2C) and was highest in the 25 °C group, followed by the 20 °C group and was lowest in the 30 °C group (all post-hoc tests *p* ≤ 0.025). We did not detect any strain specific differences in mean pup body mass (F = 3.34, *p* = 0.075; Figure 2C), and we did not notice any differences in the within litter variation in body mass depending on cage temperature (Kruskall Wallis Test: *p* = 0.389).

Finally, we found that the mean tail length of litters, when corrected for pup body mass, was affected by cage temperature (F = 45.04, *p* < 0.001; Figure 2D) and significantly increased with rising temperatures (all post-hoc tests *p* ≤ 0.035). We found no difference in the relative tail length of pups from B6D2F1 versus B6N mothers (F = 1.26, *p* < 0.267; Figure 2D).

### 3.4. Food Consumption and Amount of Faeces

**Figure 3:**
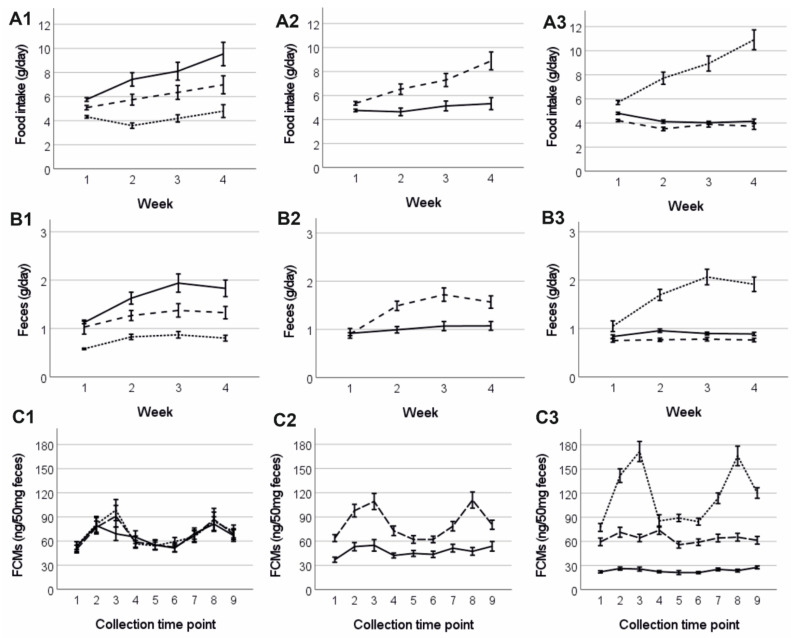
Mean (±SE) animal food consumption per day (**A1**–**A3**) and faeces production within 24 h (**B1**–**B3**) in the first, second, third and fourth week of the experiment. (**C1**–**C3**) Mean (±SE) faecal corticosterone metabolite (FCM) levels at different time points over the course of the experiment. Time points 1 and 2 were pre-birth, time point 3 was on the day of birth, time points 4–9 followed in 3–4 days intervals after birth, and the last time point was at weaning. Control mice were sampled at the same days. **A1**–**C1** shows the pooled data for mice kept at 20 °C (solid line), 25 °C (dashed line) and 30 °C (dotted line). **A2**–**C2** shows the pooled data for B6N (solid line) and B6D2F1 (dashed line) mice. **A3**–**C3** shows the pooled data for male (solid line), non-reproducing female (dashed line) and reproducing female (dotted line) mice.

When investigating animal food consumption, we found that B6D2F1 hybrid mice consumed on average significantly more food per day compared to B6N mice (F = 21.12, *p* < 0.001; Figure 3A2). Also, daily food intake was affected by cage temperature (F = 27.58, *p* < 0.001; Figure 3A1) and was significantly reduced with rising cage temperatures (all post-hoc tests: *p* ≤ 0.002). In addition, food intake also varied between mice depending on their sex and breeding status (F = 49.56, *p* < 0.001; Figure 3A3). Experimental (breeding) females consumed significantly more food compared to mice from the control groups (*p* < 0.001). This result might be slightly biased by the fact that pups in the third week also consumed some food and spillage was not taken into account. No difference was found between male and female control mice (*p* = 0.535). In line with the higher food consumption, B6D2F1 hybrids produced significantly more faeces per day than B6N mice (F = 19.48, *p* < 0.001; Figure 3B2). Moreover, faeces production significantly decreased in parallel to food consumption with rising ambient temperatures (F = 29.72, *p* < 0.001; Figure 3B1; all post-hoc tests: *p* < 0.001). Finally, daily faeces production varied between mice depending on their sex and breeding status (F = 41.76, *p* < 0.001; Figure 3B3) and breeding females produced significantly more faeces compared to mice from the control groups (*p* < 0.001). Again, no difference was observed between female and male control mice (*p* = 0.539).

### 3.5. Faecal Corticosterone Metabolites (FCMs) and Plasma Corticosterone

FCM levels differed significantly between mouse strains (F = 42.78, *p* < 0.001; Figure 3C2), as B6D2F1 mice showed constantly higher values compared to B6N mice. In addition, FCM levels differed significantly between mice depending on their sex and breeding status (F = 305.86, *p* < 0.001; Figure 3C3): Breeding females showed significantly higher FCM levels compared to both, control females and males (*p* < 0.001) and control females showed significantly higher FCM levels compared to control males (*p* < 0.001). Interestingly, breeding females showed peak values in FCM levels at the time of birth and at weaning of their offspring. However, FCM levels did not differ between mice depending on their cage temperature (F = 0.71, *p* = 0.493; Figure 3C1).

Finally, plasma corticosterone levels at the end of the experiment confirmed the findings of the FCM analysis and did not show any difference between strains (F = 0.0, *p* = 0.997) or temperature groups (F = 2.89, *p* = 0.059; data not shown).

## 4. Discussion

In our study, we investigated the effect of different housing temperatures (20 °C, 25 °C, 30 °C) on laboratory mice during the physiological challenging period of lactation and on the development of their offspring until weaning. We considered the number and body weight of weaned offspring as measurement for reproductive performance, and physiological parameters like food intake, changes in body weight and the level of stress hormones, measured by faecal corticosterone metabolites, as parameters of animal welfare status. The study included breeding and non-breeding C57BL/6N (B6N) inbred and B6D2F1 hybrid mice.

### 4.1. Reproduction

As expected from hybrid vigor, we found that pregnancy rates after four days of mating were significantly higher in B6D2F1 compared to B6N females. Neither pregnancy rate nor litter size at birth differed between experimental temperature groups, confirming that there was no bias in reproductive performance before the temperature treatment started. This result was expected, because mating and the first period of the pregnancy took place at 20 °C for all experimental females. In line with this, cage temperature and strain had no effect on the number of implantation sites. The low number of 3 pregnant B6N females out of 12 plugged after mating in the 25 °C group might be an unfortunate divergence. Although plug positive females were equally treated and randomly assigned to one of the temperature groups, the number of B6N litters was reduced for unknown reasons in females dedicated to the 25 °C group. Parameters of reproduction are, however, strongly affected by inbreeding depression. This became obvious by a generally reduced pregnancy rate of B6N females compared to hybrids in our experiment. The low number of pregnant B6N females in the 25 °C group was considered in the statistical tests.

The measured postnatal parameters litter size at weaning and mean pup body mass at weaning were significantly affected by cage temperature and reached their poorest outcome in females kept at 30 °C. Interestingly, the impact of a 30 °C cage temperature on reproduction was more pronounced in B6N females, suggesting an increased sensitivity of this inbred strain to high ambient temperatures, whereas hybrids seemed to cope better with higher temperatures. The better reproductive outcome of B6D2F1 females in all temperature groups can probably be attributed to the heterosis effect in this strain.

Our observed impact of higher ambient temperatures on reproduction is similar to results from Yamauchi and co-workers [37], who described decreased litter sizes and increased pup losses in ICR outbred mice kept at temperatures from 26 °C to 32 °C. In another study with SWISS mice, milk production at 33 °C was only 18% of that at 21 °C. This led to a reduction in pup growth by 20%, but only little pup mortality (0.8%) was observed [51]. In contrast to our study, where heat exposure started at the last third of pregnancy, Zhao and co-workers exposed females and their litters from day 6 post-partum to higher temperatures. Given that pup loss in our study occurred exclusively during the first 24 h after birth, delayed expose to temperature treatments might conceal potential temperature effects on female reproductive investment. Similar to our study, a negative temperature effect was also observed on reproductive parameters in rats kept at 33 °C [52] and hamsters kept at 30 °C [53,54]. In our study, the best reproductive outcome was found when females were kept at 25 °C, though there was little difference between 20 °C and 25 °C. This is in accordance with studies investigating the effects of ambient temperature on germ cell and embryo quality [38,55]. Sperm yield in males has been shown to decrease with rising ambient temperature but female oocyte quality was constant up to 28 °C [38]. In addition, embryo production following superovulation was best at temperatures of 24 °C and 26 °C [55]. Here, we were not interested in the effects of ambient temperature on gamete quality, but rather on breeding outcome, therefore all females were mated at the same temperature of 20 °C. 

When assessing the impact of cage temperature on female reproductive performance, it is important to consider that we housed animals, which were exposed to different temperatures in separate rooms. Housing mice in separate rooms could have affected female reproductive performance, as well as animal stress levels due to factors we could not control. However, we consider any potential room effects to be small, as rooms were side by side, highly standardized and animal husbandry was provided by the same persons. Induced by the high temperature we also observed a decrease in humidity in the warmest room, which could have further affected female reproductive performance and animal stress levels.

### 4.2. Physiological and Morphological Changes

Cage temperature also influenced other physiological and morphological parameters like body weight of lactating mothers and tail length in pups. Females kept at 30 °C were significantly lighter, compared to females at either 20 °C or 25 °C. The lower body weight at 30 °C could be explained by the significantly reduced food consumption in this group. Water consumption was not measured in this study. Therefore, we do not know if the reduced food intake was substituted by a higher water intake especially at 30 °C. In accordance with other studies [56,57,58] we found a significantly lower mean pup body mass at 30 °C compared to either 25 °C or 20 °C. Pup body mass is directly related to female body mass since the development of the mammary gland and lactation is dependent on adequate food and water intake. In addition, pup body mass can indirectly be affected by the impact of the ambient temperature on the lactating mother: According to the heat dissipation limit hypothesis, females cannot dissipate enough metabolic heat at higher ambient temperatures and therefore limit milk production, which results in reduced pup weight [54,59,60,61,62]. This hypothesis was critically discussed by Sadowska and coworkers [63]. Nevertheless, higher ambient temperatures lead to reduced mammary glands [64] and additionally to reduced energy, fat and total solids in the milk [65] resulting in reduced growth of newborn offspring until weaning. It was also shown in SWISS mice that milk energy output and suckling time were lower at 30 °C independent from the litter size [66].

We further found that pups from mothers kept at either 25 °C or 30 °C had significantly longer tails compared to pups from mothers kept at 20 °C. The finding of longer tails in mice reared at high temperatures has previously been reported [16,67]. However, a recent paper challenged the general assumption that the hairless and heavily vascularized tail of mice is an important structure for the dissipation of body heat [68]. Nevertheless, the observed elongation of the tail at this early developmental stage could be interpreted as an adaptation to get rid of body heat under high ambient temperatures. If this is the case, such plasticity was certainly facilitated by the postnatal growth period. Tail elongation as a potential heat adaption has also been detected in adult BALB/c females, which experienced high ambient temperatures from juvenile age on [15].

### 4.3. Glucocorticoids

FCM levels assessed from late pregnancy until weaning and plasma corticosterone levels at the end of the experiment did not differ between mice depending on cage temperature groups, suggesting that none of the chosen ambient temperatures was more or less stressful for the mice. Alternatively, mice might have perceived specific temperatures as stressful, but could have behaviourally adjusted to them, i.e., built a warm nest and spend more time in it at lower temperatures, or reduce their activity and try to cool at cage walls at higher temperatures. We did not conduct observations to confirm behavioural adaptations. However, we noted reduced nest building activity in the 30 °C group, which would support our assumption (see Supplementary Materials Figure S2).It is important to note that our study has some limitations in the assessment of animal stress levels. First, the relocation of mice to new rooms in only two, but not all three temperature groups might have imposed different stress levels on mice between groups, as we did not perform sham relocations. However, the increased frequency of relocations with rising temperatures could have only created a bias in our data, but has unlikely overwritten any effects of heat stress. Second, a few days prior to giving birth, females were caged individually. The handling and change in social environment might have altered female stress levels and we cannot exclude that the pre-birth faecal samples (collection time point 1 and 2, see Figure 3C1–C3) might show elevated FCM levels, rather than representing baseline value as intended. 

We found that hybrid mice showed constantly higher FCM levels compared to B6N mice. This is an interesting observation, because the observed plasma corticosterone levels in blood samples collected one day later did not show any difference between temperature groups or strains. Differences in FCM levels between strains are known from another study [40] and might be explained by genetic differences and not by differences in experienced stress levels *per se*, as both strains were treated identically. We found that FCM levels differed significantly between mice depending on their sex and breeding status. Sex differences in FCM levels are also well described [48,49] und our results confirm that males have generally lower values than females.

Not surprisingly, we further found a difference in FCM levels based on female reproductive status. Breeding females had significantly higher levels than non-breeding control females. Interestingly, breeding females showed their peak values in FCM levels at the time of birth and in the third/last week of lactation. Similar to our finding, a perinatal increase of FCM levels was also reported by Möstl and Palme [69]. It seems that birth itself, like in many other mammals, and the challenge between a decreasing milk supply at the end of the weaning period combined with an increasing food requirement in offspring is most stressful for reproducing females.

The question emerged whether more food intake and higher amounts of faeces lead to lower FCM concentrations. Studies in cows [70] and rats [71] showed that increased food intake causes a higher metabolic rate, a higher glucocorticoid clearance rate, and therefore, more FCM excretion via faeces. Interestingly, reproducing females, which consumed more food and produced more faeces, still had higher FCM levels. Therefore, the FCM concentration was not dependent on the total amount of excreted faeces and a correction in our study was not necessary.

## 5. Conclusions

It is unquestionable that ambient temperature can have a major impact on mouse physiology, from heart rate and blood pressure [7] to tumor growth [35,72,73] and immunological parameters [72,73]. Similarly, also other external factors such as humidity, microbiological status, light intensity, noise, nutrition, and others are known to have an impact [74,75,76,77]. Recently, it has been reported that adult female mice do not respond with increased excretion of stress metabolites in faeces when kept at low (20 °C) or high (30 °C) ambient temperatures [32]. Our results confirmed and extended this finding, as neither a low (20 °C) nor a high cage temperature (30 °C) resulted in significantly changed stress hormone levels in lactating mice and their non-breeding female and male controls. We conclude from our study that the ›cool‹ standard temperature in mouse facilities (21 +/− 1 °C)—with keeping conditions comparable to our experimental conditions—has no negative effect on animal stress levels, as long as nest building material is provided. In contrast, we found that a comparatively high ambient temperatures of 30 °C can reduce the reproductive performance and induce specific anatomical and physiological changes in mothers and their offspring (i.e., increased tail length, reduced body weight). We are aware that our study has specific limitations as discussed above, but in consideration of our findings, we cannot recommend a homeothermic cage temperature of 30 °C for breeding mice. Independent of animal welfare aspects, room temperatures of around 30 °C can be challenging for employees working tightly dressed in a mouse facility [38,78].

## Figures and Tables

**Table 1:** Number of parturient B6N and B6D2F1 females per plug positive females that were kept at 20 °C, 25 °C and 30 °C.

	20 °C	25 °C	30 °C
**B6N**	7/13	3/12	9/12
**B6D2F1**	11/13	11/12	11/12

## Data Availability

No new data sets were created or analyzed in this study. Data sharing is not applicable to this article.

## Supplementary Materials

The following supporting information can be downloaded at: https://www.mdpi.com/article/10.3390/ani12162141/s1, Table S1: Temperature within cages and humidity within rooms (mean ± SD) at the three temperature levels, Figure S1: Examples of lactating B6N (a,c) and B6D2F1 (b,d) females in the third week at 20 °C (a,b) and 30 °C (c,d), Figure S2: Examples of cages with B6N (a,c) and B6D2F1 (b,d) pups in the third week at 20 °C (a,b) and 30 °C (c,d).
