# Supplementary material for: Effect of Different Ambient Temperatures on Reproductive Outcome and Stress Level of Lactating Females in Two Mouse Strains
Source: Animals (Basel). 2022 Aug 20;12(16):2141. doi: 10.3390/ani12162141 (PMC9405067; doi:10.3390/ani12162141)
Supplement: Supplementary file 1 [file animals-12-02141-s001.zip › Supplementary Table S1.pdf]

		20°C	25°C	30°C
temperature	mean	19.8	25.0	31.4
	SD	0.7	0.6	1.3
humidity	mean	48.1	38.7	36.5
	SD	2.2	1.2	5.1
