# Supplementary figures and images for: Effect of Different Ambient Temperatures on Reproductive Outcome and Stress Level of Lactating Females in Two Mouse Strains
Source: Animals (Basel). 2022 Aug 20;12(16):2141. doi: 10.3390/ani12162141 (PMC9405067; doi:10.3390/ani12162141)

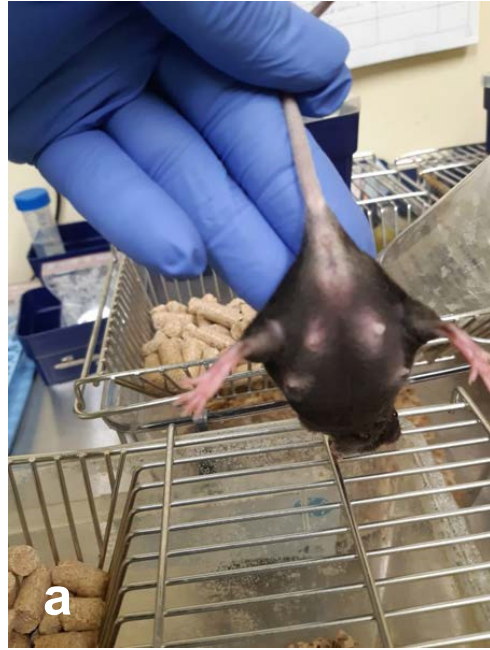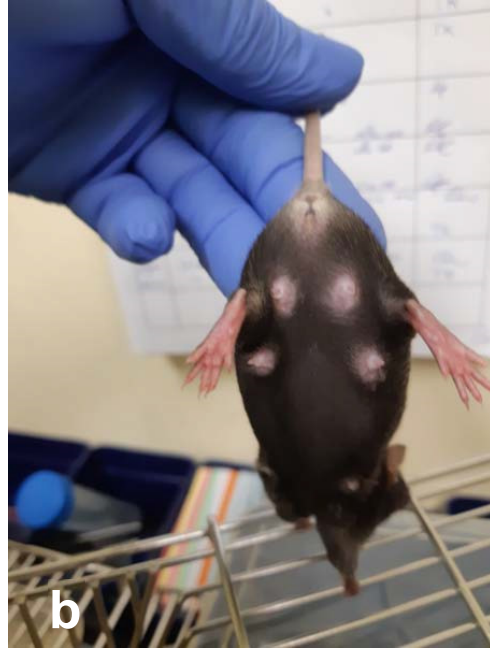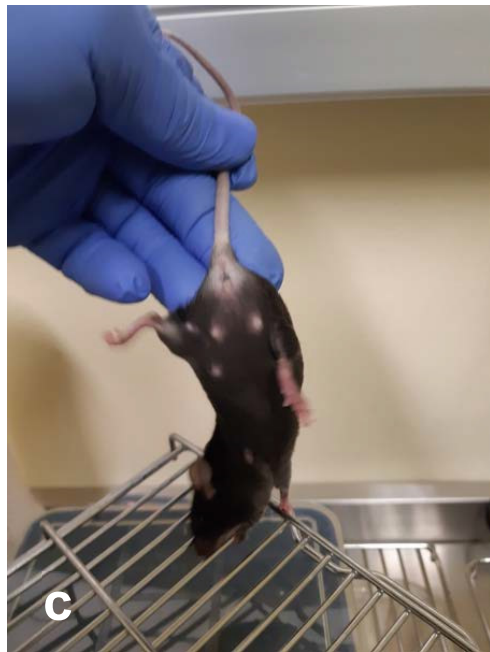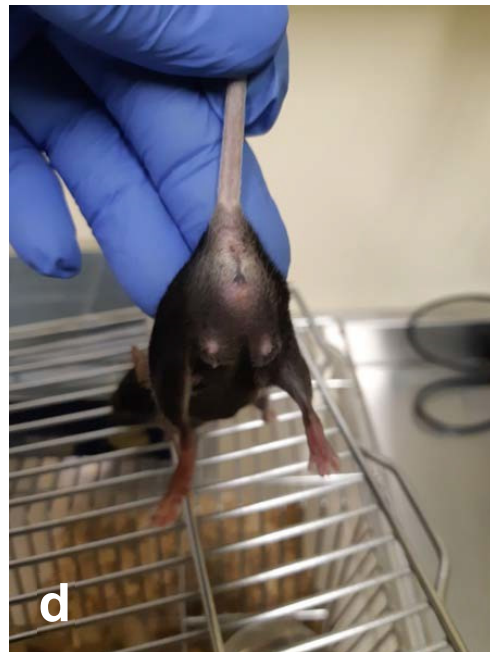

Supplement: Supplementary file 1 [file animals-12-02141-s001.zip › Supplementary Figure S1.pdf]

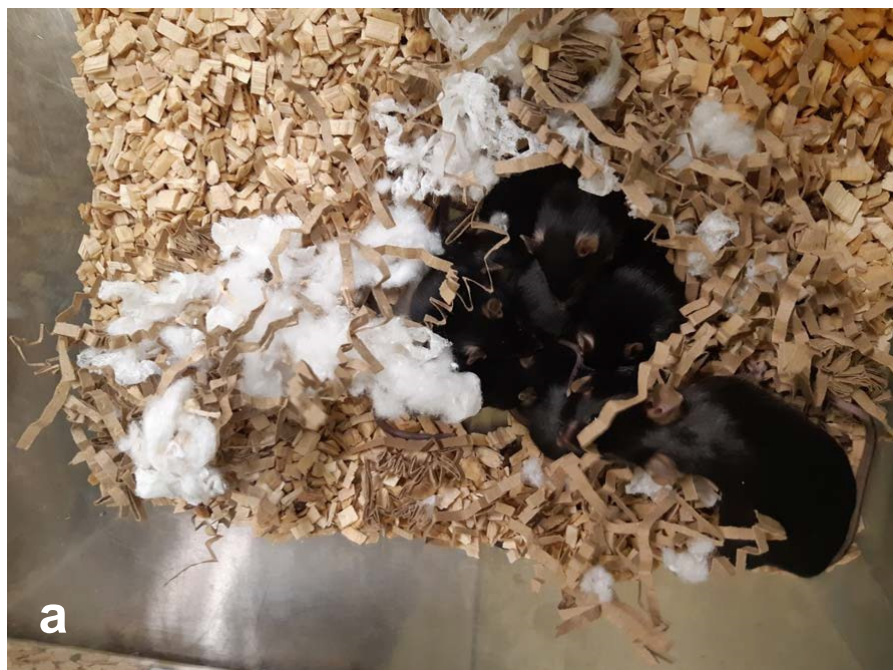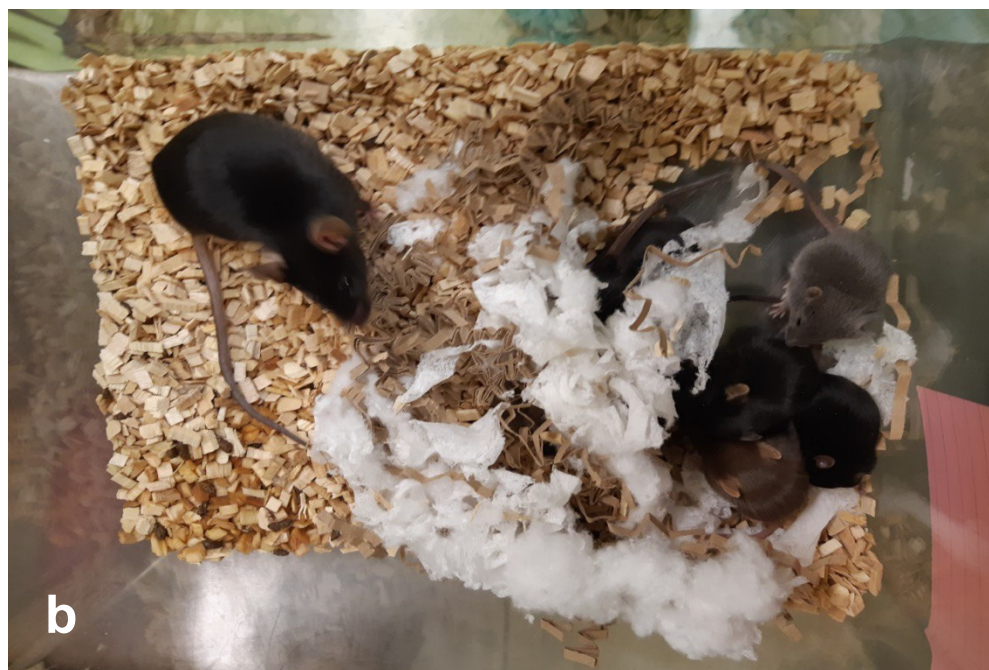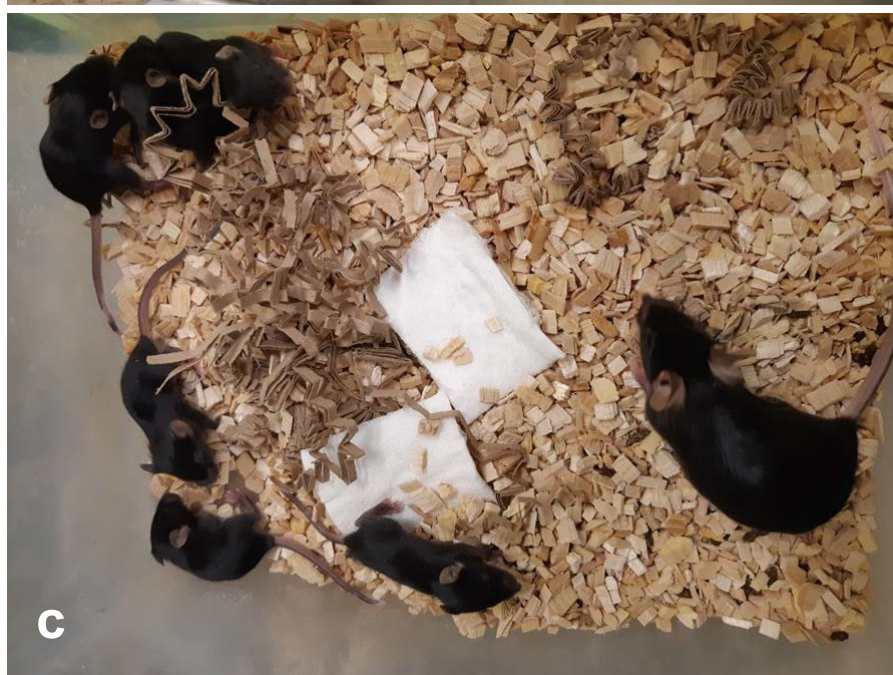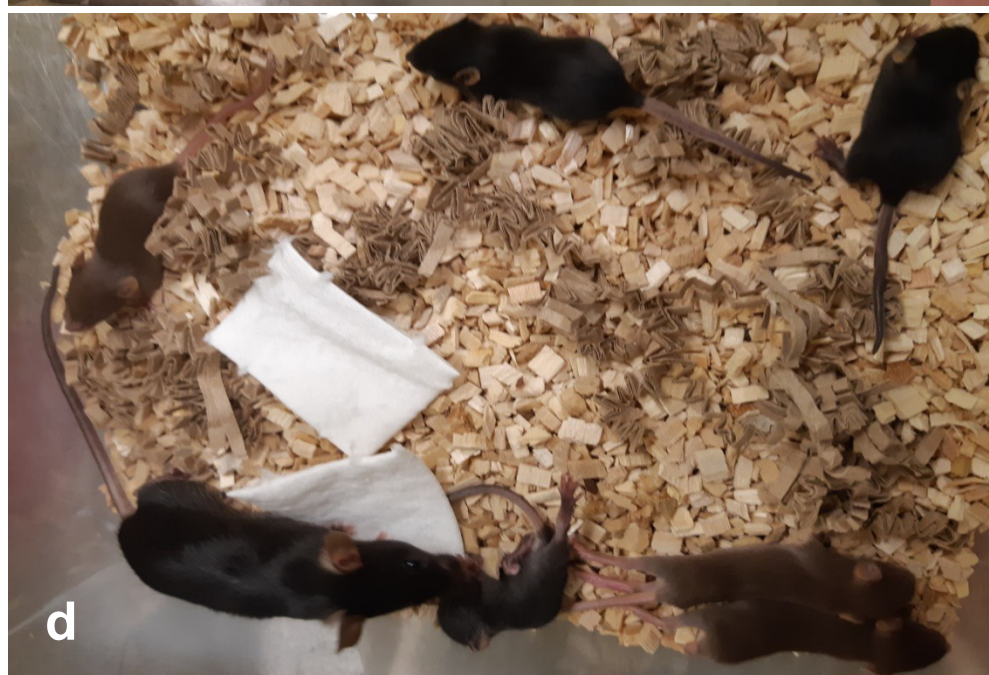

Supplement: Supplementary file 1 [file animals-12-02141-s001.zip › Supplementary Figure S2.pdf]
